# Rapid Standardized CT-Based Method to Determine Lean Body Mass SUV for PET—A Significant Improvement Over Prediction Equations

**DOI:** 10.3389/fonc.2022.812777

**Published:** 2022-07-07

**Authors:** Terence A. Riauka, Vickie E. Baracos, Rebecca Reif, Freimut D. Juengling, Don M. Robinson, Marguerite Wieler, Alexander J. B. McEwan

**Affiliations:** ^1^ Division of Medical Physics, Department of Oncology, University of Alberta, Edmonton, AB, Canada; ^2^ Division of Palliative Care Medicine, Department of Oncology, University of Alberta, Edmonton, AB, Switzerland; ^3^ Division of Oncologic Imaging, Department of Oncology, University of Alberta, Edmonton, AB, Canada; ^4^ Medical Faculty, University Bern, Bern, Switzerland; ^5^ Department of Physical Therapy, University of Alberta, Edmonton, AB, Canada

**Keywords:** LBM, SUV, SUL, PET, PET/CT

## Abstract

In ^18^F-fluorodeoxyglucose (^18^F-FDG) positron emission tomography (PET) studies, maximum standardized uptake value (SUV_max_) is the parameter commonly used to provide a measurement of the metabolic activity of a tumor. SUV normalized by body mass is affected by the proportions of body fat and lean tissue, which present high variability in patients with cancer. SUV corrected by lean body mass (LBM), denoted as SUL, is recommended to provide more accurate, consistent, and reproducible SUV results; however, LBM is frequently estimated rather than measured. Given the increasing importance of a quantitative PET parameter, especially when comparing PET studies over time to evaluate disease response clinically, and its use in oncological clinical trials, we set out to evaluate the commonly used equations originally derived by James (1976) and Janmahasatian et al. (2005) against computerized tomography (CT)-derived measures of LBM.

**Methods:**

Whole-body ^18^F-FDG PET images of 195 adult patients with cancer were analyzed retrospectively. Representative liver SUV_mean_ was normalized by total body mass. SUL was calculated using a quantitative determination of LBM based on the CT component of the PET/CT study (LBM_CT_) and compared against the equation-estimated SUL. Bland and Altman plots were generated for SUV-SUL differences.

**Results:**

This consecutive sample of patients undergoing usual care (men, n = 96; women, n = 99) varied in body mass (38–127 kg) and in Body Mass Index (BMI) (14.7–47.2 kg/m2). LBM_CT_ weakly correlated with body mass (men, r^2^ = 0.32; women, r^2^ = 0.22), and thus SUV and SUL_CT_ were also weakly correlated (men, r^2^ = 0.24; women, r^2^ = 0.11). Equations proved inadequate for the assessment of LBM. LBM estimated by James’ equation showed a mean bias (overestimation of LBM compared with LBM_CT_) in men (+6.13 kg; 95% CI 4.61–7.65) and in women (+6.32 kg; 95% CI 5.26–7.39). Janmahasatian’s equation provided similarly poor performance.

**Conclusions:**

CT-based LBM determinations incorporate the patient’s current body composition at the time of a PET/CT study, and the information garnered can provide care teams with information with which to more accurately determine FDG uptake values, allowing comparability over multiple scans and treatment courses and will provide a robust basis for the use of PET Response Criteria in Solid Tumors (PERCIST) in clinical trials.

## Introduction

Optimal therapeutic management of cancer patients is dependent upon accurate diagnosis, staging, and the assessment of both short- and long-term treatment efficacy. To this end, 18F-fluorodeoxyglucose (^18^F-FDG) positron emission tomography (PET) has become an integral component in the modern standard of cancer care (1–3). The introduction of PERCIST has emphasized the opportunity offered to the oncology community by this metabolic imaging methodology (4, 5). Quantification of the uptake of ^18^F-FDG in tissue is expressed in terms of standard uptake value (SUV) as defined by:


(1) $$\text{SUV}=\frac{\mathrm{Activity}\;\text{concentration}\left(\frac{kBq}{ml}\right)}{\frac{\mathrm{injected}\;\mathrm{dose}\;\left(MBq\right)}{\text{total}\;\text{body}\;\text{mass }\left(kg\right)}}$$

This total body mass (TBM)-based SUV (SUVTBM) fails to take into account the current understanding of the effect of body composition (i.e., the proportions of lean and fat tissue in the body) on measured activity concentrations of ^18^F-FDG in tissue and the variability of body composition that occurs during a patient’s cancer journey.

A precise and specific analysis of body composition is accessible using computed tomography (CT) images acquired during routine care (6) and is becoming a major area of cancer research. Muscle mass or lean body mass (LBM) is increasingly seen as an important factor in predicting cancer outcomes including mortality, treatment toxicity, and complications of cancer surgery (7, 8). As of January 2021, more than 580 publications and 30 meta-analyses have reported on CT-defined body composition in oncology patients (8). The potential impact of wide variations in body composition on optimum dosing of anticancer drugs has been discussed in relation to toxicity and treatment efficacy (9–11). The impact of body composition on SUV in ^18^F-FDG-PET/CT is not negligible mainly due to the fact that ^18^F-FDG uptake occurs primarily in lean tissues (12). Fat contributes to overall body mass but accumulates minimal ^18^F-FDG [i.e., 2%–25% of SUV in lean tissues (12)]; fat can thus obfuscate the clinical utility of SUVTBM, therefore limiting its effectiveness for diagnosis, staging, and management of patients with cancer. Obese patients are suggested to be particularly at risk for overestimation of the activity of both normal tissues and malignant lesions when TBM is used to determine the amount of injected activity (13). Patients who have identical weight, and therefore the same injected dose of ^18^F-FDG, can have a multiplicity of SUV depending on their proportions of body fat and lean tissue. This being the case it is little wonder that some have taken to referring to the SUV as a “silly useless value” (14). Replacing TBM with LBM has been proposed to result in a more rigorous assessment of ^18^F-FDG uptake (SUL) in reference and diseased tissues (13, 15).


(2) $$SUL=\frac{\mathrm{activity}\;\text{concentration}\left(\frac{kBq}{ml}\right)}{\frac{\mathrm{injected}\;\mathrm{dose}\;\left(MBq\right)}{\mathrm{lean}\;\mathrm{body}\;\mathrm{mass}\;\left(kg\right)}}=SUV\cdot \frac{LBM}{TBW}$$

Dual-energy x-ray absorptiometry (DXA) is regarded as a reference method for the estimation of human body composition including fat mass, fat-free mass, and bone mineral mass (16). Estimation of LBM may be generated from the equations of James (17) and Janmahasatian et al. (18) (Janma):


(3) $${\text{LBM}}_{\text{James}}=\{\begin{array}{c} \left(1.07\cdot W\right)-148\cdot {\left(\frac{W}{H}\right)}^{2}\;\text{Females}\\ \left(1.1\cdot W\right)-128\cdot {\left(\frac{W}{H}\right)}^{2}\;\text{Males} \end{array}$$

(4) $${\text{LBM}}_{\text{Janna}}=\{\begin{array}{l} \frac{9.27\cdot 10^{3}\cdot W}{8.78\cdot 10^{3}+244\left(\frac{W}{h^{2}}\right)}\;\text{Females}\\ \frac{9.27\cdot 10^{3}\cdot W}{6.68\cdot 10^{3}+216\left(\frac{W}{h^{2}}\right)}\;\text{Males} \end{array}$$

where *W* is weight in kilograms (kg), *H* is height in centimeters (cm), and *h* is height in meters (m) for the individual patient. These equations are not identical, so SUV for a given patient will depend on which estimate of LBM (James or Janmahasatian) is used. More importantly, neither equation was based on LBM data from patients with cancer. Janmahasatian’s equations were formulated on data from 303 healthy individuals with a mean age of 41 years, recruited from the students and staff of a university (17); James’ 56 subjects (mean age 60 years) were recruited from two specialist obesity clinics (18). Despite questionable applicability to cancer patients, most recent publications use either James’ or Janmahasatian’s equations to develop SUL values (15, 19). The body habitus of contemporary cancer patients ranges from underweight to morbidly obese and includes wide variations in composition including highly skewed compositions such as sarcopenic obesity (7, 20, 21). This intrinsic variability is increased if we take into account the primary site, the stage of disease, and the treatment status. Patient-specific measurements of LBM are required to eliminate estimation-related bias on SUL. CT scans have been shown to be effective at precisely quantifying patient-specific LBM in patients with solid tumors using the total cross-sectional area of muscle at the L3 vertebral body, as verified by dual-energy x-ray (r = 0.94) analysis (6). These CT scan data are intrinsically available for patients with cancer who undergo CT and/or PET/CT scanning as part of their clinical workup and subsequent follow-up and can be used to provide individualized determinations of LBM. These determinations should result in more accurate diagnosis, planning for most appropriate treatments, and the assessment of treatment efficacies, including the recognition of treatment futility. Patient-specific CT-based LBM-^18^F-FDG uptake (SULCT) values may be generated accordingly.


(5) $$SUL_{CT}=\frac{\mathrm{activity}\;\text{concentration}\left(\frac{kBq}{ml}\right)}{\frac{\mathrm{injected}\;\mathrm{dose}\;\left(MBq\right)}{\text{CT}-\mathrm{based}\;\text{lean}\;\text{body}\;\text{mass}}}$$

## Materials and Methods

This study was approved by the Human Research Ethics Board of Alberta–Cancer Care (HREBA.CC.15.0057). Two hundred oncological patients (100 consecutive men and 100 consecutive women) scheduled for routine clinical PET/CT at the Cross Cancer Institute (CCI), the regional cancer center for northern Alberta, Canada (population 2 million), were included in this study. Of these, four men and one woman were excluded due to extensive liver disease that precluded accurate normal liver SUV determination. Normal liver SUV and LBM_CT_ were determined retrospectively for the included patients.

Patients were required to fast for 6 h prior to their PET/CT appointment. Blood glucose monitoring (values between 4 and 10 mmol/l were considered acceptable) was performed prior to ^18^F-FDG injection. PET/CT, using oral contrast (15 ml Omnipaque 300 in 1 L of water), was performed using a Siemens Biograph mCT scanner (TRUEV model, 21.6 cm axial field of view; Siemens Healthineers USA, Inc., Knoxville, TN, USA). The injected activity was 5.2 MBq/kg of ^18^F-FDG, and PET scans were acquired at 60 ± 10 min post injection. Patients were positioned in the scanner head-first, supine, with arms up if capable. CT scanning was performed immediately prior to the PET scan using the following parameters (120 kVp/50 mA-Care Dose4D; slice 1.5 mm; pitch 0.7; rotational speed 0.5 s). PET scan acquired emission data for 2 min per bed position, and for all patient scans, coverage was, at minimum, from the base of the brain to mid-thigh. PET data were corrected for randoms, dead time, scatter, and attenuation and then reconstructed iteratively using ordered subset expectation maximization algorithm (3 iterations, 24 subsets) and a post reconstruction 2-mm Gaussian filter was applied. PET reconstruction slice matrix was 200 × 200 with 2-mm slice thickness.

Axial CT slices landmarked at the third lumbar vertebra were examined to determine the total cross-sectional area (cm^2^) of the following muscles: *rectus abdominis*, abdominal (*transversus abdominis*, *abdominal external oblique*, *abdominal internal oblique*), *psoas*, and paraspinal *(quadratus lumborum and erector spinae)*. At this level, muscle cross-sectional area is linearly related to whole-body LBM as determined by dual-energy x-ray (6):


(6) LBM=0.03×SMA+6.06

where LBM is measured in kg and SMA is the skeletal muscle area determined by CT at L3 in cm^2^ (r = 0.94, p < 0.001, standard error 0.72, mean residual error 2.94 kg).

Muscle segmentation was performed anatomically within a prespecified Hounsfield range of -29 to +150 HU utilizing Slice-O-Matic software (v.4.3 Tomovision, Magog, QC, Canada) (6). Using these data, LBM_CT_ was determined for each patient and compared with TBM. For comparison to LBM_CT_, LBM_James_ and LBM_Janma_ were also calculated.

SUV_mean_ and SUL_mean_ for liver were determined for each patient using a 1.5-cm radius spherical volume of interest placed in a central portion of the liver containing no lesions. SUV_TBM_ were calculated according to Equation 1. Correlation analysis was conducted, and *r* ^2^ values for correlation plots were reported. Bland and Altman analysis (22) was conducted to evaluate the degree of agreement between the CT-defined measures of LBM and the values given by the two equations. Bland and Altman analysis is performed by studying the mean difference between the two measures and by constructing the limits of agreement. This analysis is considered more robust than correlation to assess agreement (23). This was a preliminary study. We did not have any *a priori* knowledge for choosing the sample size.

All statistics and figures presented here were created using R: A Language and Environment for Statistical Computing (version 4.0.3, R Foundation for Statistical Computing, Vienna, Austria) (24) and the following libraries: ggplot2 (25), ggpp (26), ggmisc (27), and blandr (28).

## Results

**Figure 1:**
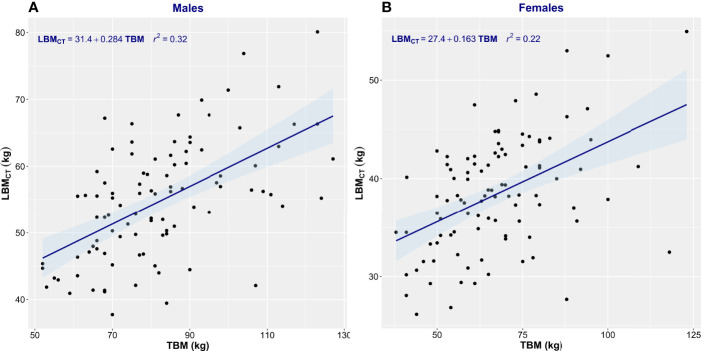
Scatter plot of total body mass (TBM) vs. computed tomography–defined lean body mass (LBM_CT_). Men **(A)** and women **(B)**. The linear regression equation is provided in the top left of each graph, and it is plotted in blue. The shaded light-blue area represents the 95% confidence interval, and *r* ^2^ is the square of the Pearson correlation coefficient for TBM vs. LBM_CT_.

**Figure 2:**
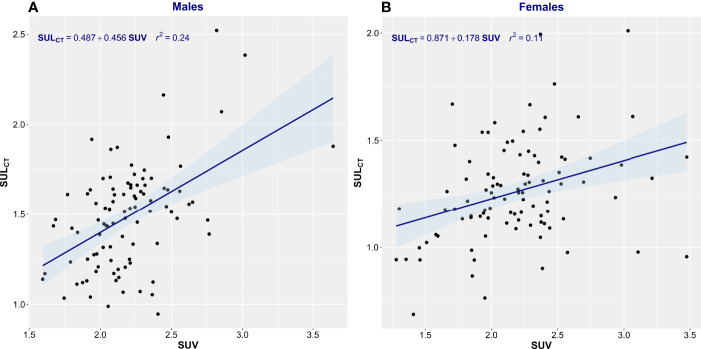
Scatter plot of standardized uptake value (SUV) vs. standardized uptake adjusted by lean body mass (SUL_CT_). Men **(A)** and Women **(B)**. The linear regression equation is provided in the top left of each graph, and it is plotted in blue. The shaded light-blue area represents the 95% confidence interval, and *r* ^2^ is the square of the Pearson correlation coefficient for SUV vs. SUL_CT_.

Patient characteristics are summarized in **Table 1**. This consecutive sample receiving standard cancer care showed variation in TBM from 38 to 127 kg and in BMI from 14.7 to 47.2 kg/m^2^, spanning severely underweight to morbidly obese. Fat mass (4.3–68 kg) and body fat percentage (8.3%–60.2%) were the most variable features. Body composition is sex-dependent, so all results are shown separately for male and female subjects. LBM_CT_ weakly correlated with TBM in both sexes (men, *r* ^2^ = 0.32; women, *r* ^2^ = 0.22) (**Figure 1**). **Figure 1** demonstrates the substantial differences that can exist between CT-determined LBM among individuals of identical body mass. For example, men of 70 ± 1 kg TBM had LBM_CT_ ranging from 37.7 to 56.0 kg and a fat mass ranging from 13.4 to 27.6 kg. This degree of variability was evident across the entire range of TBM values and, as a consequence, SUV and SUL_CT_ were weakly correlated [men, *r* ^2^ = 0.24; women, *r* ^2^ = 0.11 (**Figure 2**)]. Taken together, these results suggest that body composition is so variable that it renders SUV based on body mass uninterpretable.

**Table 1:** Patient characteristics.

Characteristic	Men (n = 96)	Women (n = 99)	p-value
Age (years)	59.3 ± 13.8	59.7 ± 15.5	0.498
Weight, kg [mean, SD (range)]	82.2 ± 17.5 [52–127]	67.4 ± 16.6 [38–123]	<0.001
Total fat cross-sectional area, cm^2^ [mean, SD (range)]	347.2 ± 197.0 [2.49–926.2]	318.9 ± 191.4 [17.6–939.1]	0.309
Body mass index, kg/m^2^ [mean, SD (range)]	27.0 ± 5.4 [17.6–44.5]	26.0 ± 6.1 [14.7–47.3]	0.113
Total skeletal muscle cross-sectional area, cm^2^ [mean, SD (range)]	162.3 ± 29.2 [105.6–246.8]	107.9 ± 19.2 [67.0–162.9]	<0.001
Fat mass, kg [mean, SD (range)]	27.8 ± 13.5 [4.3–67.1]	25.8 ± 13.0 [5.3–68.0]	0.309
Body fat percentage [mean, SD (range)]	32.4 ± 11.1 [8.3–53.8]	36.4 ± 12.0 [12.3–60.2]	0.002
Lean body mass, kg [mean ± SD (range)]			
Computed tomography	54.8 ± 8.8 [37.7–80.1]	38.4 ± 5.8 [26.1–54.9]	<0.001
Estimated by James’ equation	60.9 ± 7.5 [43.2–78.0]	44.7 ± 5.4 [32.4–59.2]	<0.001
Estimated by Janmahasatian’s equation	60.4 ± 7.7 [42.9–78.5]	40.8 ± 6.2 [28.5–60.6]	<0.001

**Figure 3:**
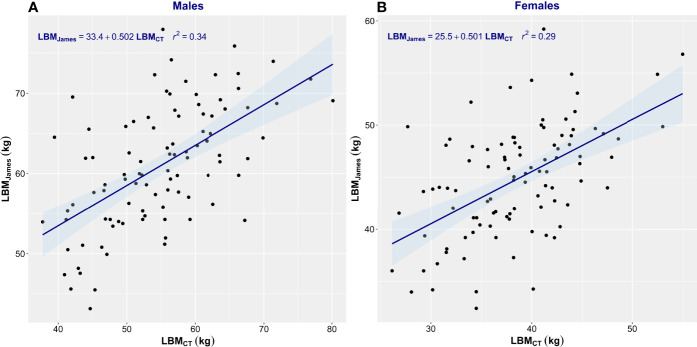
Scatter plot of CT-defined lean body mass (LBM_CT_) vs. that estimated by James’ equation (LBM_James_). Men **(A)** and women **(B)**. The linear regression equation is provided in the top left of each graph, and it is plotted in blue. The shaded light-blue area represents the 95% confidence interval, and *r* ^2^ is the square of the Pearson correlation coefficient for LBM_CT_ vs. LBM_James_.

**Figure 4:**
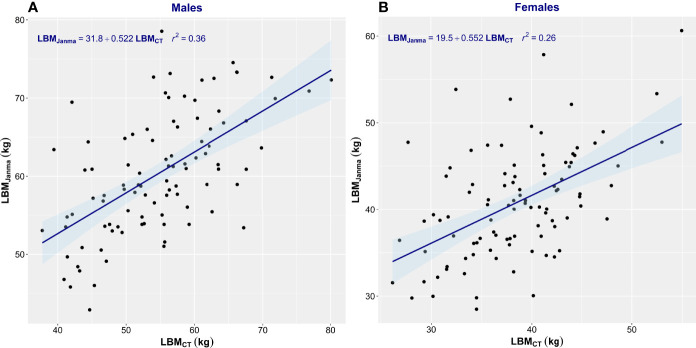
Scatter plot of CT-defined lean body mass (LBM_CT_) vs. that estimated by Janmahasatian’s equation (LBM_Janma_). Men **(A)** and women **(B)**. The linear regression equation is provided in the top left of each graph, and it is plotted in blue. The shaded light-blue area represents the 95% confidence interval, and *r* ^2^ is the square of the Pearson correlation coefficient for LBM_CT_ vs. LBM_Janma_.

Estimation equations proved inadequate for the assessment of LBM. The correlation between LBM_CT_ and that estimated by James’ equation was low (men, *r* ^2^ = 0.34; women, *r* ^2^ = 0.29) (**Figure 3**). James’ equation most often overestimated LBM (82% of men and 87% of women) but also underestimated LBM in 18% of men and 13% of women (**Table 2**). LBM estimated by Janmahasatian’s equation gave similarly poor performance as with the James equation. While not identical, both equations are based merely on height and weight, so the performance metrics of the two equations were similar. The correlation between LBM_CT_ and that estimated by Janmahasatian’s equation was low (men, *r* ^2^ = 0.36; women, *r* ^2^ = 0.26) (**Figure 4**). This equation also usually overestimated LBM in 80% of men and 66% of women but also underestimated LBM in 20% of men and 34% of women (**Table 2**).

**Table 2:** Overestimation and underestimation of lean body mass (LBM) by the use of equations.

	Underestimation (LBM_equation_/LBM_CT_) < 1				Overestimation (LBM_equation_/LBM_CT_) > 1			
	Men	Women	Men	Women	Men	Women	Men	Women
% of patients	18%	13%	20%	34%	82%	87%	80%	66%
	LBM_James_/LBM_CT_		LBM_Janma_/LBM_CT_		LBM_James_/LBM_CT_		LBM_Janma_/LBM_CT_	
Minimum	0.81	0.84	0.79	0.75	1.01	1.01	1.01	1.01
Maximum	0.98	0.99	0.99	0.99	1.65	1.80	1.65	1.73
Mean	0.93	0.94	0.93	0.92	1.17	1.21	1.16	1.15
Median	0.93	0.95	0.93	0.93	1.13	1.18	1.13	1.09

**Figure 5:**
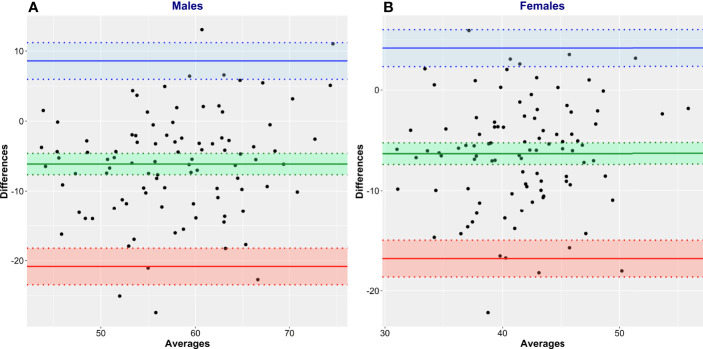
Bland and Altman plots of CT-defined lean body mass (LBM_CT_) vs. that estimated by James’ equation (LBM_James_). LBM_CT_ is the reference method, and LBM_James_ is the test method. Men **(A)** and women **(B)**.

**Figure 6:**
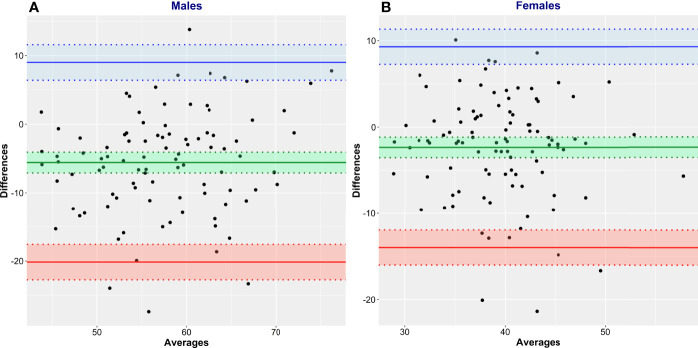
Bland and Altman plots of CT-defined lean body mass (LBM_CT_) vs. that estimated by Janmahasatian’s equation (LBM_Janma_). LBM_CT_ is the reference method, and LBM_Janma_ is the test method. Men **(A)** and women **(B)**.

Bland and Altman analysis was conducted to further evaluate the degree of (dis)agreement between LBM_CT_, the reference method, and the values given by James’ equation, the test method (**Figure 5**, **Table 3**). LBM estimated by James’ equation showed a mean bias (overestimation of LBM compared with LBM_CT_) in men of +6.13 kg (95% CI 4.61–7.65) and in women of +6.32 kg (95% CI 5.26–7.39) (**Table 3**). Limits of agreement were very large (**Table 3**), e.g., for men, the upper limit of agreement was +20.84 kg (95% CI 18.23–23.45) and the lower limit of agreement was -8.59 kg (95% CI 5.98–11.20), spanning an absolute limit of agreement of 29.43 kg. LBM estimated by Janmahasatian’s equation, the test method, showed a mean bias (overestimation of LBM compared with LBM_CT_, the reference method) in men of +5.58 kg (95% CI 4.07–7.49) and in women of +2.34 kg (95% CI 1.15–3.52) (**Figure 6**, **Table 3**).

**Table 3:** Bias and upper and lower limits of agreement between CT-defined lean body mass (LBM_CT_) and LBM estimated by the equations of James and Janmahasatian (LBM_James_, LBM_Janma_).

Sex	Men	Men	Women	Women
Equation	James	Janma	James	Janma
Reference	CT	CT	CT	CT
n	96	96	99	99
Significance level	0.95	0.95	0.95	0.95
Significance level to z	1.96	1.96	1.96	1.96
Bias, kg	6.13	5.58	6.32	2.34
Bias Upper CI	4.61	4.07	5.26	1.15
Bias Lower CI	7.65	7.09	7.39	3.52
Bias SD	7.51	7.44	5.34	5.94
Bias SEM	0.77	0.76	0.54	0.60
Limit of Agreement (LOA)				
LOA SEM	1.31	1.30	0.92	1.02
Lower LOA [CI]	-8.59 [-11.20, -5.98]	-9.00 [-11.58, -6.41]	-4.25 [-5.98, -2.32]	-9.30 [-11.33, -7.27]
Upper LOA [CI]	20.84 [18.23, 23.45]	20.16 [17.57, 22.74]	16.80 [14.97, 18.63]	13.97 [11.94, 16.00]

## Discussion

In this sample, we observed a 3-fold range of body mass and 7-fold range in body fat percentage. The evidence presented here supports the conclusion that body composition is highly variable in a population-based cohort of patients presenting for ^18^F-FDG-PET/CT studies at a comprehensive cancer center. This variability is not represented by the measurement of overall body weight. These findings are in accordance with prior studies (7, 10, 20, 21) and underscore the large diversity in proportion of lean tissue and adipose tissue in the human body in patients with cancer.

Variability in body proportions of lean tissue and fat has important implications for any injected substance that tends to partition mainly in the lean (or fat) compartments (29). Many cancer drugs are scaled to body mass or to body surface area (BSA, also calculated from height and weight); however, heterogeneous body composition may contribute to interpatient variation in pharmacokinetics of antineoplastic agents and their toxicities. Specifically, LBM has been suggested (9–11) to be of particular relevance for anticancer agents that distribute in and are metabolized within the lean compartment. Distribution/uptake in lean tissue is also a feature of ^18^F-FDG (12). Injected ^18^F-FDG activity is, however, most often based on TBM, as patients with larger TBM attenuate a larger fraction of the emitted 511 keV photons and, therefore, require more activity to produce images of diagnostic quality. Unfortunately, TBM clearly fails to properly represent the tissue compartment in which ^18^F-FDG uptake occurs, and as such, the discrepancy between conventional body weight-based SUV and SUL_CT_ in this dataset is impressive. Without a patient-specific LBM determination, the portion of TBM composed of fat remains an unknown, resulting in erroneous SUV. Utilizing the full potential of SUV becomes difficult when the true body composition of individuals is unknown.

Attempting to predict LBM using the James and Janmahasatian equations fails to yield accurate values in adult patients with cancer. Indeed, the notion of an “average” body composition is implausible given the extent of the variation typically observed in clinical populations (7, 10, 20, 21). Both equations systematically overestimate LBM. This is not unexpected given that these equations were originally developed with data from young healthy subjects who are likely to be more muscular and have less adipose tissue compared with patients with cancer who are typically older and ill. While a general trend may be identified between objectively determined and estimated LBM, the significant bias and the scatter in the data (i.e., wide limits of agreement) render accurate patient-specific determinations of LBM using the James and Janmahasatian equations untenable.

Determining LBM_CT_ allows for a patient-specific evaluation of the amount of tissue that will accumulate ^18^F-FDG. When CT data are not available prior to the injection of activity, this information may be obtained from the CT portion of a PET-CT scan from which LBM_CT_ correction factors may be derived. CT-based measures of LBM allow for patient-specific determinations of ^18^F-FDG uptake based on an accurate assessment of an individual’s true amount of tissue for which activity accumulation is pertinent. Previous proposals for CT-based measures of LBM included whole-body segmentation using a 4-compartment model (bone, soft tissue, fat, and air) (30). The proposal presented here uses a simple extensively validated approach based on the delineation of four unequivocally defined muscle groups at the level of the third lumbar vertebra, which can be performed by the automated software and subsequently edited by a trained user in less than 6 min.

The specific approach that we used relies on the high correlation (r = 0.94) between muscle cross-sectional area in the lumbar region and whole-body lean mass by DXA (6), and this aligns with the similarly high correlation between muscle and fat areas in the lumbar region and whole-body muscle and fat mass (31). This extrapolation to whole-body values is robust and gets around the challenge that the head and the legs are not scanned completely in typical whole‐body PET/CT. CT-based approaches have been proposed by others (30, 32–34) using different strategies: Either was lean mass estimated indirectly as body weight - CT-defined fat mass, which introduces a measurement error for the body weight value and the CT fat mass (i.e., voxels within a range of attenuation of −190 to −30 Hounsfield units, assumed to have an average density of 0.923 g/ml), where the imputed fat mass values were not validated against DXA. In a further approach, comprehensive whole-body segmentation using a 4-compartment model (bone, soft tissue, fat, and air) (30) had been performed, which is both computational extensive and again prone to bias introduced by tissue definition based on partially overlapping Hounsfield unit definitions. Subsequently, these approaches have not reached clinical routine.

We propose that LBM_CT_ provides a robust measure of SUV that can be quickly performed and incorporated into the patient data reconstruction workflow to provide LBM-corrected SUVs at the time of clinical reporting and will inform a response criterion such as PERCIST in a manner that will provide more consistency in the measurement and hence more clinical value as a marker of response and potential outcomes. As metabolic imaging is increasingly incorporated into management algorithms and is increasingly used in clinical trials, there is a recognized need to create more robust and more standardized measurements of SUV (4, 30, 32–34), including accurate measures rather than estimates of SUL.

## Conclusions

Deriving patient-specific LBM and SUL_CT_ is readily available using CT data obtained during routine PET/CT scans. Performing an LBM correction to weight-based SUVs should provide a more clinically robust measure of FDG uptake in normal and diseased tissues. Furthermore, SUL_CT_ values will not be biased by patient body composition changes over time, as they inherently incorporate the patient’s current body composition at the time of the scan. Since a CT scan is performed with every PET/CT study, the information garnered can provide care teams with information with which to more accurately determine FDG uptake values. Clinical measures based on FDG uptake, such as total lesion glycolysis (TLG) and metabolic tumor volume (MTV), should also be less affected by body composition changes with time and, therefore, provide more robust measures for tracking/evaluating disease state. Using CT-based evaluations of LBM on a routine basis would allow for SUL_CT_ use in PERCIST 1.0, providing a more accurate measure of clinical response to treatment decisions.

## Data Availability Statement

The raw data supporting the conclusions of this article will be made available by the authors without undue reservation.

## Ethics Statement

The studies involving human participants were reviewed and approved by the Human Research Ethics Board of Alberta–Cancer Care (HREBA.CC.15.0057). Written informed consent for participation was not required for this study in accordance with the national legislation and the institutional requirements.

## Author Contributions

TR and VB have contributed equally to this work and share first authorship. All authors listed (TR, VB, RR, FJ, DR, MW and AM) have made a substantial, direct, and intellectual contribution to the work and approved the submitted version for publication.

## Funding

VB: Canadian Institutes of Health Research, Alberta Cancer Foundation.

## Conflict of Interest

The authors declare that the research was conducted in the absence of any commercial or financial relationships that could be construed as a potential conflict of interest.

## Publisher’s Note

All claims expressed in this article are solely those of the authors and do not necessarily represent those of their affiliated organizations, or those of the publisher, the editors and the reviewers. Any product that may be evaluated in this article, or claim that may be made by its manufacturer, is not guaranteed or endorsed by the publisher.
